# Correction

**DOI:** 10.1080/15685551.2020.1795509

**Published:** 2020-08-27

Article title: Synthesis of Some Silyl Mono- and Polystyrenes with New Properties

**Authors**: Assadi, M. G., & Hosseinzadeh, F.

**Journal**: *Designed Monomers and Polymers*

**Citation details**: Volume 13, Number 2, pages 181–191

**DOI**: https://doi.org/10.1163/138577210X12634696333433

**Article title**: Synthesis and Characterization of Some Quaternized Styrene Monomers and Polymers

**Authors**: Galehassadi, M., Herizchi, R., & Ranjbar, F.

**Journal**: *Designed Monomers and Polymers*

**Citation details**: Volume 14, Number 4, pages 303–313

**DOI**: https://doi.org/10.1163/138577211X577178

The university name for the authors’ affiliation was misspelled in the above articles. The corrected affiliation for the authors is:

Department of Chemistry, Azarbaijan University of Tarbiat Moallem, Tabriz, Iran

